# Interpupillary Distance and Binocular Vision: Assessing the Association Between Anatomy and Sensory-Motor Functions

**DOI:** 10.3390/brainsci16040401

**Published:** 2026-04-09

**Authors:** Mosaad Alhassan, Mona Aljami, Rakan Alotaibi, Muhamad Alrashed, Balsam Alabdulkader, Wafa Alotaibi, Tahani Alqahtani, Madhwi Aldhwayan, Ali Almustanyir

**Affiliations:** 1 Department of Optometry, College of Applied Medical Sciences, King Saud University, P.O. Box 10219, Riyadh 11433, Saudi Arabia; monaaljami2@gmail.com (M.A.); 441100871@student.ksu.edu.sa (R.A.); mrashed@ksu.edu.sa (M.A.); alabdulkader@ksu.edu.sa (B.A.); walotaibi@ksu.edu.sa (W.A.); talqahtani@ksu.edu.sa (T.A.); aalmustanyir@ksu.edu.sa (A.A.); 2 Department of Community Health Sciences, College of Applied Medical Sciences, King Saud University, Riyadh 11433, Saudi Arabia; maldhwayan@ksu.edu.sa

**Keywords:** interpupillary distance, near point of convergence, phoria, stereopsis, binocular vision, ergonomics

## Abstract

**Highlights:**

**What are the main findings?**

- Wider interpupillary distance impairs convergence and stereopsis at distance.
- Interpupillary distance selectively affects binocular vision, not horizontal phoria.
- Near point of convergence strongly predicts distance stereopsis.

**What are the implications of the main findings?**

- Anatomical variation in IPD significantly influences sensory-motor visual performance, with clinical implications for binocular vision assessment.
- IPD values at the extremes of the distribution may increase visual demands during near-work tasks, informing ergonomic and clinical management strategies.

**Abstract:**

**Background/Objectives**: This study aimed to examine how interpupillary distance (IPD) influences motor and sensory binocular vision functions at distance and near, and to examine associations between different motor and sensory binocular vision functions. **Methods**: A descriptive cross-sectional study was performed involving 100 random participants of both genders, aged 20 to 38 years. Optically corrected and non-strabismic participants were selected from King Saud University, Riyadh, Saudi Arabia between June 2024 and June 2025. Distance and near IPD were assessed with a pupillometer. Motor binocular functions included horizontal phoria using the von Graefe technique and near point of convergence (NPC). Sensory binocular vision was assessed using crossed and uncrossed TNO and Random-dot E stereopsis tests at near and distance respectively. Statistical analysis was conducted using SPSS version 26 (*p* ≤ 0.05). **Results**: IPD and phoria were not significantly associated at distance or near. However, moderate and significant correlations were found between IPD (distance and near) and NPC. Significant correlations were also found between IPD and both crossed and uncrossed stereopsis at distance, but correlations were weak or non-significant at near. A strong correlation was observed between NPC and stereopsis at distance. **Conclusions**: As interpupillary distance (IPD)increases, near point of convergence (NPC) receded, and stereopsis at distance declined, suggesting that anatomical variation influences binocular vision primarily at distance.

## 1. Introduction

Binocular vision is the main key to providing full and comprehensive view of the world. In simple terms, binocular vision refers to the ability to use both eyes simultaneously to perceive a single three-dimensional image [1,2]. Normal binocular vision requires integration between sensory functions (i.e., visual perception), and proper oculomotor functions (i.e., eye movement). In normal binocular vision, each eye perceives a similar but slightly different image due to their different horizontal position in the human head. The two images are then combined into one single image. The process of combining the two images into one image is classified, according to Worth, into three grades [3]. The first grade is simultaneous perception which is the elementary level of binocularity. Simultaneous perception refers to the ability of the two eyes to perceive similar but horizontally separated images at the same time; each one is formed on the corresponding retinal area. The second grade is fusion which is defined as the unification of the two visual stimuli from the corresponding retinal areas into a single image, and it is classified as sensory and motor fusion. Sensory fusion is the ability to appreciate two similar images, one with each eye, and interpret them as one. Motor fusion is the ability to align both eyes so the sensory fusion can be maintained over wide range of distances. The third grade is stereopsis which is the ability to perceive depth and three-dimensional objects by fusing the slightly different images represented by each eye [3].

Abnormal binocular vision encompasses a variety of conditions that can result from different causes and etiologies. Abnormal binocular vision mainly occurs when the two eyes are not properly aligned on the same object of regard. This misalignment between the two eyes could lead to further complications such as vergence dysfunctions [4]. Previous studies reported high rates of vergence anomalies among populations [4,5,6,7]. Different types of vergence dysfunctions are strongly associated with ocular symptoms such as eye strain, headache, blurred vision, and double vision especially after prolonged near work [8,9].

There are a number of clinical examinations that can be used to assess sensory and motor functions of binocular vision. Most common motor clinical examinations are heterophoria and near point of convergence (NPC). Heterophoria, or simply phoria, is a clinical test that measures the accuracy of horizontal and vertical ocular alignment of the two eyes when focusing on an object of regard at both distance and near, and it is measured in prism diopter. It can present as an inward deviation (esophoria), outward deviation (exophoria), or vertical deviation (hyperphoria and hypophoria). Small degrees of heterophoria are common in visually normal individuals. Near point of convergence (NPC) refers to the closest point at which the visual axis intersects and converges with the maximum effort while still maintaining binocular single vision [10,11]. Stereopsis is a sensory binocular function test that refers to the ability of the visual system to fuse the two images from the two eyes, resulting in the perception of depth or three-dimensional structure [10,11]. Stereopsis can be measured clinically by presenting two similar targets, one for each eye, using polaroid lenses or red-green filters. Stereopsis threshold is the minimum angular separation between the two targets that a person can detect, and it is measured in seconds of arc. Depth can be appreciated when the two visual axes intersect in front of the plane of the targets—this is called crossed disparity stereopsis. Also, depth can be appreciated when the two visual axes intersect behind the plane of the targets—this is called uncrossed disparity stereopsis [12]. Stereopsis tests can be categorized based on the type of stimulus into contour (local stereopsis), and random-dot (global stereopsis). Contour tests use simple shapes to extract depth. However, depth perception using random-dot stimulus is a more complex process requiring accurate integration between visual information from the two eyes and higher visual centers [13].

Inter-pupillary distance (IPD) is the distance between the centers of the pupils. IPD has been found to be crucial and beneficial in various fields, including anatomy, genetics, prosthodontics, and craniofacial surgeries. In optometry, IPD is essential for positioning ophthalmic lenses; the optical center of each lens must align with the center of the pupils to avoid unwanted prismatic effects, which can cause visual disturbances such as discomfort, image distortion, and diplopia [14,15]. IPD varies across different populations and must be accurately measured to ensure proper spectacle and other ocular device manufacturing [16].

Inter-individual variability in sensory and/or motor binocular vision measurements can be attributed to a range of factors. These include functional differences in the ciliary muscle and lens, other optical influences, and psychological elements like an individual’s subjective criterion for blur. Furthermore, experimental error introduces additional noise into the measurements [17]. Variations in facial anatomical features may also contribute to variations in sensory and motor binocular vision. For example, interpupillary distance (IPD) that falls outside the normal range, often due to an individual’s unique facial anatomy, may impair ocular alignment and stereopsis. Despite this, the specific relationship between IPD and sensory and motor binocular vision parameters remains poorly understood.

There is limited and conflicting evidence in the literature regarding the correlation between horizontal phoria and IPD. Some studies have reported a weak correlation between the two variables, which appears to depend on various factors. Generally, individuals with extremely large IPD values tend to exhibit some degree of exophoria, while those with very small IPD values may exhibit some degree of esophoria [14]. While NPC and IPD are different measurements, some correlation may exist between them. Previous studies suggest a positive correlation, as larger IPD values typically require greater convergence effort to rotate the eyes inward. Conversely, smaller IPD values may require less convergence effort due to the anatomically closer position of the eyes [18]. With respect to stereopsis, previous studies focused on associations between crossed disparity contour stereopsis at near with IPD. In general, association was very weak but as IPD is getting wider, stereopsis declined [19]. This study aims to investigate the association between IPD with motor binocular vision functions (horizontal phoria and NPC) and sensory binocular vision functions (crossed and uncrossed random-dot stereopsis) at both distance and near. To our knowledge, this is the first study to investigate variations in IPD on both motor and sensory binocular vision functions at distance and near. Association between different motor and sensory binocular vision measurements will be investigated as well.

## 2. Materials and Methods

### 2.1. Subjects

This study was conducted at the Department of Optometry, College of Applied Medical Sciences, King Saud University, Riyadh, Saudi Arabia between June 2024 and June 2025. The study was approved by the ethical committee at King Saud University Medical City (Ethics Number 24/1317/IRB). Each participant signed an informed consent form to participate in the study after receiving information on the objectives of the research.

Healthy young adults aged between 20 and 38 years old and with optically corrected monocular visual acuity of at least 6/6 at distance were recruited. Participants with a history of any systemic disease such as diabetes or hypertension, previous ocular surgery or any ocular disease such as diabetic retinopathy, corneal opacity, cataract, or glaucoma, and those who take medications known to affect visual functions were excluded from the study. One hundred participants were recruited (52 females, 48 males). The difference in age between the two groups is not statistically significant, and the two groups were pooled into one group for further analysis.

### 2.2. Procedures

The study started by measuring preliminary visual tests and performing comprehensive ocular examinations to determine eligibility to participate. The examinations included the best optically corrected monocular and binocular visual acuity at distance using logMAR chart, objective refraction measurements by auto-refractometer (NIDEK ARK-510A, Gamagori, Aichi, Japan), and slit lamp examinations. After that, the clinical examinations related to the objective of the study were measured. First, IPD was measured at near and distance using a pupillometer (Topcon PD-5 Pupilometer, Topcon Healthcare, Oakland, NJ, USA). Second, motor binocular vision measurements included near point of convergence (NPC) and horizontal phoria. NPC was measured using the RAF ruler, and the amount of horizontal phoria was measured at 40 cm and 6 m, according to the von Graefe technique, using a phoropter. Finally, stereopsis was measured to evaluate sensory binocular vision functions. Crossed and uncrossed stereopsis were measured at distance and near, using two random-dot stereo tests: TNO test (Alfred P. Poll Inc., New York, NY, USA) at near, and Random Dot-E test (Stereo Optical Co., Inc., Chicago, IL, USA) at distance. Table 1 lists the details of stereopsis tests and their major features. All measurements were taken three consecutive times, and the average values were recorded. The design, methods and clinical procedures of all tests are described in the previous literature [10,11,20].

### 2.3. Data Analysis

All variable data are expressed in numerical values and entered into SPSS version 26 for further analysis. Normal distribution for all variables was tested using the Shapiro–Wilk Test. Results showed IPD is normally distributed (*p* = 0.213). Phoria, NPC, and stereopsis are not normally distributed (Phoria, *p* = 0.049; NPC and stereopsis tests *p* < 0.001). Association between independent variables (IPD at distance and near) and dependent variables (Phoria, NPC, and stereopsis) was tested by Spearman’s Rank Correlation (ρ) Test. Association between motor tests (Phoria and NPC) and sensory tests (stereopsis) was also tested by Spearman’s Rank Correlation (ρ) Test. If the correlation test showed significant association, then linear regression was conducted. Significance level was set at *p* ≤ 0.05.

Participants were also classified based on the amount of horizontal phoria into three groups: orthophoric (between 2 Base-out prism diopters to 2 Base-in prism diopters), esophoric (>2 Base-out prism diopters), and exophoric (>2 Base-in prism diopters). IPD at distance and near, and NPC values were compared across groups using one-way ANOVA. Pairwise multiple comparisons according to the Bonferroni method were applied. ANOVA on Rank test was used to examine differences between groups in stereopsis values at distance and near.

## 3. Results

### 3.1. Descriptive Statistics

Table 2 reports mean, standard deviation, range, minimum, and maximum values for continuous variables: IPD, NPC, and phoria. Table 3 reports median, minimum, maximum, and interquartile ranges for ordinal variables: stereopsis.

### 3.2. Correlation Between IPD and Motor Function Tests

**Figure 1:**
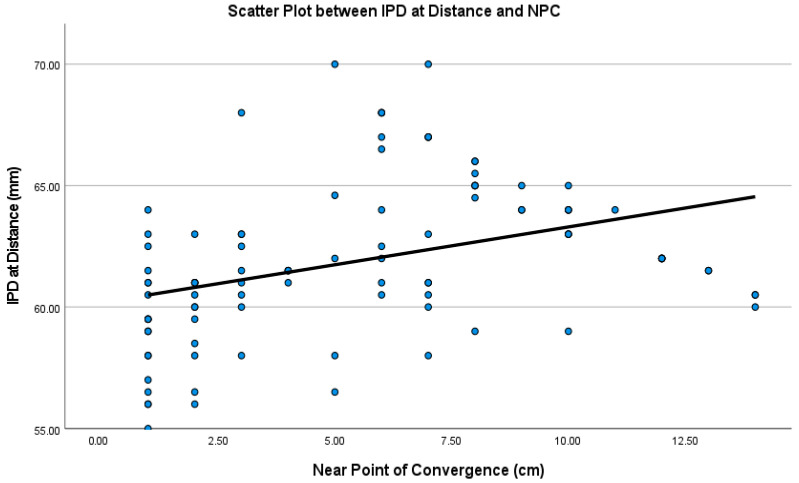
Scatter plot showing linear regression between IPD at distance and near point of convergence.

Table 4 summarizes the results of Spearman correlation coefficients and linear regression between IPD at distance and near, with the amount of horizontal phoria at distance and near, and NPC. Results showed that there is no significant correlation between IPD and phoria at distance, and there is a weak negative correlation between IPD and phoria at near. However, the linear regression did not show significant linearity between the two variables. Interestingly, both IPD values at distance and near showed moderate and significant correlation with NPC. Also, the two variables showed linear relationships: as the IPD value increases, the NPC increases as well (i.e., gets further away). Figure 1 shows a scatter plot between IPD at distance and NPC. A similar relationship exists with IPD value at near.

### 3.3. Correlation Between IPD and Sensory Function Tests

Table 5 summarizes the results of Spearman correlation coefficients and linear regression between IPD at distance and near with crossed and uncrossed stereopsis. Results showed there is a significant correlation between IPD and both crossed and uncrossed stereopsis at distance. Linear regression tests showed significant linearity between the two variables: as the IPD value got wider, both crossed and uncrossed stereopsis declined at distance. There is a weak negative correlation between IPD and crossed stereopsis at near; however, the linear regression did not show significant linearity. Results showed no significant correlation between IPD and uncrossed stereopsis at near.

### 3.4. Correlation Between Motor and Sensory Function Tests

Table 6 summarizes the results of correlation and linear regression between the amount of phoria and NPC with stereopsis at distance and near. Strong, positive, and significant correlation and regression was observed between NPC and both crossed and uncrossed stereopsis at distance: as convergence ability reduces (i.e., receded NPC), stereopsis declines as well. There was a weak correlation between phoria at near with crossed stereopsis only.

### 3.5. Comparisons of Different Types of Phoria as a Function of IPD, NPC, and Stereopsis

**Figure 2:**
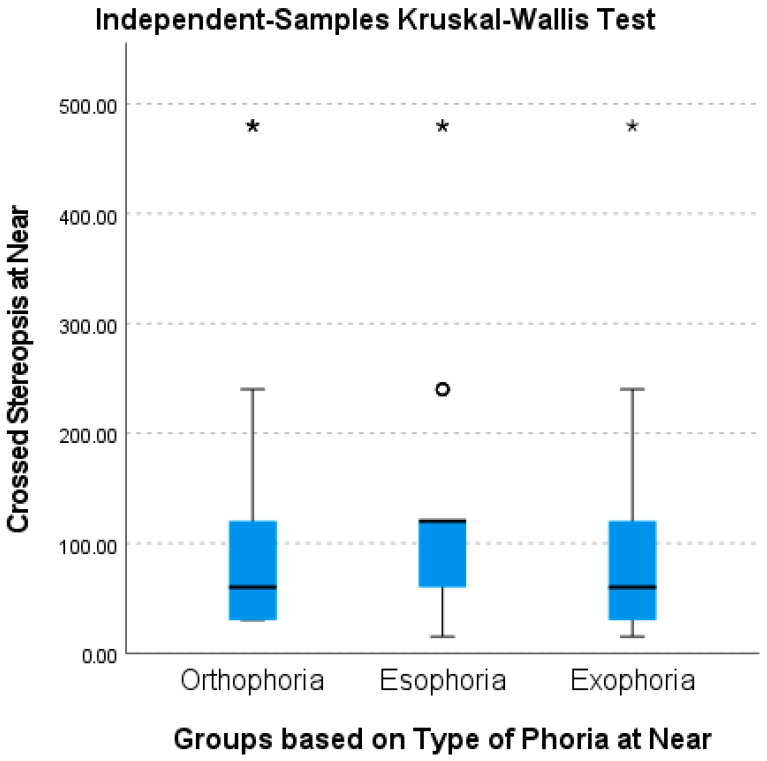
Box-plot chart of subjects with different types of phoria as a function of crossed disparity TNO stereopsis test at near. Vertical bars represent the 10–90 percentile range. The box represents the 25–75% quartiles. Solid horizontal lines represent medians. Circles indicate outliers. Star signs represent maximum disparity for this test (480 s of arc).

Table 7 and Table 8 show the comparisons of IPD, NPC, and stereopsis with individuals of different types of phoria at distance and near. Results for phoria at distance did not reveal any significant differences with all types of tests. Unexpectedly, orthophoric participants at near showed a further (receded) NPC than both exophoric and esophoric ones by more than 3 cm on average. With respect to stereopsis tests at near, esophoric participants had worse stereopsis than both orthophoric and exophoric ones. The differences between esophoric and both orthophoric and exophoric participants reached significance for the crossed disparity test (Figure 2). For the uncrossed disparity test, differences were significant between esophoric and exophoric, but not with orthophoric ones.

## 4. Discussion

The aim of this study is to assess the association between anatomical features of the location of the two eyes in the head, represented by IPD, with different motor and sensory binocular vision functions at both distance and near. Results found no significant association between IPD and horizontal phoria at distance. A weak negative correlation between IPD and near phoria was observed, although linear regression analysis did not confirm statistical linearity, indicating that as IPD increases, individuals tend to be more exophoric at near. However, this finding is not conclusive and requires a larger sample size with all ranges of IPD to be confirmed. Absence of association between IPD and phoria is supported by mean IPD values across phoria groups. Previous studies found conflicting results: Darko-Takyi et al. found no significant correlations between IPD and phoria [21], while Hall and colleagues found that individuals with extremely large IPD values tend to exhibit exophoria, and those with very small IPD values may exhibit esophoria at near [18].

On the contrary to the relationship between IPD and phoria, the present study revealed a significant and direct correlation between IPD and NPC at both distance and near. This means the maximum ability to converge our eyes becomes more difficult as IPD values increase. This is consistent with findings of previous studies reporting remote NPC values among those with larger IPDs [15,21]. These results suggest that maximum convergence ability rather than ocular alignment (i.e., phoria) is influenced by IPD. As IPD values increase, the angular disparity between the two eyes increases as well, which could lead to extra effort on the medial recti muscles to converge to maximum amplitude in order to maintain bifoveal fixation during near tasks. These results suggest the importance of considering IPD values when determining normative values of NPC in clinical settings.

With respect to the influence of IPD on sensory binocular vision functions (i.e., stereopsis), results found significant correlation between the two variables at distance only. Both crossed and uncrossed stereoacuity decreased by the same amount as IPD increases. Correlation was either very weak or not significant at near. Iqbal et al. found significant correlation between larger IPD and worse stereopsis [19]; however, another study found the opposite [22]. Conflicting results across different studies may be attributed to variations in methodology and stereopsis stimulus. The conflicting results shown in this study between association of IPD with stereopsis at distance and near may be due to different factors. First, the horizontal disparity between the two eyes is smaller at distance compared to near, which may result in difficulties in fusing the two images at distance as IPD increases. Also, the accommodation–vergence relationship at near may provide strong feedback for optimal binocular fusion.

With respect to the correlation between motor and sensory binocular vision functions, results found a significant correlation between NPC and both crossed and uncrossed stereopsis at distance. As convergence ability reduces (i.e., receded NPC), stereopsis worsens as well. A previous study found similar findings between the two variables at near [23]. Relationships between phoria and stereopsis were not significant most of the time. The significant association between NPC and stereopsis at distance rather than near agrees with IPD effects on NPC and stereopsis at distance only, suggesting that binocular vision at distance is more likely to be affected by anatomical variations, while binocular vision at near remains relatively independent.

When comparing different binocular vision functions according to magnitude of ocular alignment (i.e., phoria), results revealed unexpected findings. Orthophoric participants demonstrated receded NPC by around 3 cm compared to both exophoric and esophoric individuals. Also, crossed disparity stereopsis at near was significantly reduced among esophoric participants. These results suggest that deficits in NPC and stereopsis are totally independent from the magnitude of phoria. Phoria may not be a good predictor for other binocular vision functions, as most individuals can compensate for this misalignment by reserve fusional vergence. Thus, it is important when assessing binocular vision to examine all clinical measurements rather than relying solely on the magnitude of phoria. Previous studies showed mixed results when predicting different binocular vision functions based on amplitude of phoria [24,25,26].

Results of this study suggest the influence of anatomical features, represented by IPD, on the motor-sensory pathway of binocular vision. It appears that IPD (the anatomical factor) influences the maximum ability to converge the eyes and maintain binocular single vision (motor function measured by NPC), which subsequently challenges stereoscopic depth perception (sensory function) at distance only. The lack of influence of phoria on other binocular vision functions reflects that small ocular misalignment may not be of clinical importance if compensated by fusional vergence amplitude. Further studies are needed to explore whether IPD, NPC, and stereopsis are affected in individuals with high magnitude decompensated phoria with low fusional vergence amplitude. The overall results suggest certain clinical approaches when it comes to binocular vision assessment and management; for example, it would be possible that both motor and sensory functions improve when applying vision therapy training to patients with receded NPC such as convergence insufficiency (CI) patients. Further studies are required to investigate whether vision therapy training can improve both convergence ability and stereoscopic vision.

## 5. Conclusions

Results of this study establish the influence of anatomical determinants on both motor and sensory binocular vision functions. The significant correlations between the three components—IPD, NPC, and stereopsis—suggest that individuals with larger IPD place greater demand on the vergence system. This could result in reduced convergence ability (i.e., higher NPC values) and worse stereopsis, especially at distance. The weak correlations between horizontal phoria and other measurements indicate that neuromotor mechanisms and vergence adaptation effectively compensate for anatomical variations across individuals. These results confirm that full and comprehensive binocular vision assessment should include anatomical, motor, and sensory functions rather than isolated components.

## Figures and Tables

**Table 1:** Main features of clinical stereopsis tests.

Test	Type of Stereopsis	Testing Distance	Disparity Range (s of Arc)	Filters for Dichoptic Presentation	Light Level	Stimulus
TNO	Random-dot	40 cm	15–480	Red-Green	280 lx	Complex
Random Dot-E	Random-dot	4.5 m	52–504	Polaroid	280 lx	Complex

**Table 2:** Descriptive statistics for continuous variables.

Test	Mean	Standard Deviation	Range	Minimum	Maximum
IPD at Distance (mm)	61.91	3.22	15	55	70
IPD at Near (mm)	58.91	3.22	15	52	67
Near Point of Convergence (cm)	5.53	3.83	13	1.00	14
Phoria at Distance (Prism Diopter)	0.19	2.94	23	−15	8
Phoria at Near (Prism Diopter)	1.32	6.01	29	−14	15

**Table 3:** Descriptive statistics for ordinal variables (stereopsis, seconds of arc).

	Crossed Stereopsis at Near	Uncrossed Stereopsis at Near	Crossed Stereopsis at Distance	Uncrossed Stereopsis at Distance
Median	60	60	168	168
Minimum	15	15	52	52
Maximum	480	480	504	504
25th Percentile	37.50	60	83	94.75
50th Percentile	60	60	168	168
75th Percentile	120	120	252	252

**Table 4:** Correlation and linear regression results between IPD and motor binocular vision functions.

Independent Variables	Dependent Variables												
	Phoria D					Phoria N					NPC		
	Correlation (ρ)		Regression			Correlation (ρ)		Regression			Correlation (ρ)	Regression	
			β	R				β	R			β	R
IPD D	−1.00										0.47 **	0.442	0.37 **
IPD N						−0.21 *		−0.34	−0.185		0.47 **	0.448	0.37 **

D: Distance, N: Near, (ρ): Spearman correlation coefficient, β: Regression constant, R: Regression coefficient, (*) *p* value is statistically significant at 0.05 level, (**) *p* value is statistically significant at 0.01 level.

**Table 5:** Correlation and linear regression results between IPD and sensory binocular vision functions.

Independent Variables	Dependent Variables					
IPD D	Crossed Stereopsis at Distance			Uncrossed Stereopsis at Distance		
	Correlation (ρ)	Regression		Spearman (ρ)	Regression	
		β	R		β	R
	0.503 **	20.56	0.452 **	0.505 **	19.45	0.450 **
IPD N	Crossed Stereopsis at Near			Uncrossed Stereopsis at Near		
	Correlation (ρ)	Regression		Correlation (ρ)	Regression	
		β	R		β	R
	−0.222 *	1.464	0.042	−0.171		

D: Distance, N: Near, (ρ): Spearman correlation coefficient, β: Regression constant, R: Regression coefficient, (*) *p* value is statistically significant at 0.05 level, (**) *p* value is statistically significant at 0.01 level.

**Table 6:** Correlation and linear regression results between motor and sensory binocular vision functions.

Independent Variables	Dependent Variables					
Phoria D	Crossed Stereopsis at Distance			Uncrossed Stereopsis at Distance		
	Correlation (ρ)	Regression		Correlation (ρ)	Regression	
		β	R		β	R
	−0.047			−0.057		
Phoria N	Crossed Stereopsis at Near			Uncrossed Stereopsis at Near		
	Correlation (ρ)	Linear Regression		Correlation (ρ)	Linear Regression	
		β	R		β	R
	0.258 **	1.238	0.066	0.177		
NPC	Crossed Stereopsis at Distance			Uncrossed Stereopsis at Distance		
	Correlation (ρ)	Regression		Correlation (ρ)	Regression	
		β	R		β	R
	0.66 **	20.52	0.538 **	0.65 **	19.480	0.537 **
NPC	Crossed Stereopsis at Near			Uncrossed Stereopsis at Near		
	Correlation (ρ)	Regression		Correlation (ρ)	Linear Regression	
		β	R		β	R
	0.044			0.035		

D: Distance, N: Near, NPC: Near Point of Convergence, (ρ): Spearman correlation coefficient, β: Regression constant, R: Regression coefficient, (**) *p* value is statistically significant at 0.01 level.

**Table 7:** Mean values, standard error of the mean, 95% confidence interval, and ANOVA test for comparisons between phoria groups as a function of IPD and NPC.

Independent Variables		Mean	Std. Error	95% Confidence Interval for Mean		One-Way ANOVA	
				Lower Bound	Upper Bound	F (df)	Pairwise Multiple Comparisons (Bonferroni)
IPD D	Ortho D, (N = 69)	62.11	0.382	61.34	62.87	0.927, 2	
	Eso D, (N = 15)	60.86	0.43498	59.93	61.79		
	Exo D, (N = 16)	62	1.08	59.69	64.30		
NPC	Ortho D, (N = 69)	5.78	0.44	4.89	6.67	1.554, 2	
	Eso D, (N = 15)	6	1.28	3.23	8.76		
	Exo D, (N = 16)	4	0.72	2.45	5.54		
IPD N	Ortho N, (N = 46)	59.51	0.468	58.56	60.45	2.298, 2	
	Eso N, (N = 35)	58	0.437	57.11	58.88		
	Exo N, (N = 19)	59.11	0.92	57.15	61.06		
NPC	Ortho N, (N = 46)	7.15	0.635	5.87	8.43	8.839, 2 **	Ortho vs. Eso = 3.037 ** Ortho vs. Exo = 2.094 *
	Eso N, (N = 35)	4.11	0.472	3.15	5.07		
	Exo N, (N = 19)	4.21	0.614	2.91	5.50		

D: Distance, N: Near, NPC: Near Point of Convergence, Ortho; Orthophoria, Eso: Esophoria, Exo: Exophoria, (*) *p* value is statistically significant at 0.05 level, (**) *p* value is statistically significant at 0.01 level.

**Table 8:** Median values and ANOVA on Rank test for comparisons between phoria groups as a function of stereopsis.

Independent Variables		Median	ANOVA on Rank	
			F, df	Pairwise Multiple Comparisons
X Stereopsis D	Ortho D, (N = 69)	168	4.22, 2	
	Eso D, (N = 15)	103		
	Exo D, (N = 16)	110.50		
UX Stereopsis D	Ortho D, (N = 69)	168	0.381, 2	
	Eso D, (N = 15)	118		
	Exo D, (N = 16)	141.50		
X Stereopsis N	Ortho N, (N = 46)	60	6.875, 2 *	Eso vs. Ortho = 14.085 * Eso vs. Exo = 17.77 *
	Eso N, (N = 35)	120		
	Exo N, (N = 19)	60		
UX Stereopsis N	Ortho N, (N = 46)	60	6.04, 2 *	Eso vs. Exo = 18.97 *
	Eso N, (N = 35)	120		
	Exo N, (N = 19)	60		

X: Crossed, UX: Uncrossed, D: Distance, N: Near, Ortho: Orthophoria, Eso: Esophoria, Exo: Exophoria, (*) *p* value is statistically significant at 0.05 level.

## Data Availability

The data presented in this study are available on request from the corresponding author. The data contains other information that will be used for other studies. The corresponding author decided to not share the data for privacy and novelty of the other study.
